# Computed Tomography (CT)-Guided Needle Biopsy of Lung Lesions: A Single Center Experience

**DOI:** 10.3390/healthcare12131260

**Published:** 2024-06-25

**Authors:** Oluebube C. Ezenagu, Gaby E. Gabriel, Sibu P. Saha

**Affiliations:** College of Medicine, University of Kentucky, Lexington, KY 40506, USA

**Keywords:** CT-guided needle biopsy, lung lesions, pulmonary lesions, transthoracic needle biopsy, diagnostic rate, complication rate

## Abstract

(1) **Objective**: Lung cancer is one of the leading causes of cancer death among men and women across the globe. The accurate and timely diagnosis of lung lesions is of paramount importance for prognosis. This single-center study is the first to assess the diagnostic yield and complication rate of a computed tomography (CT)-guided needle biopsy of pulmonary parenchymal and pleural nodules in an academic training center in the United States. (2) **Methods**: This is a retrospective study approved by IRB. Patients who underwent CT-guided needle biopsy between 2016 and 2020 were reviewed. A CT-guided needle biopsy involving mediastinal lesions was excluded, focusing only on lung parenchymal and pleural lesions. A CT-guided needle biopsy aborted at any point during the procedure was also excluded from this study. (3) **Results**: 1063 patients were included in this study; 532 were males, and 531 were females. Lesion size ranged from 0.26 cm to 9.2 cm. 1040 patients received diagnoses, among which 772 had a specific diagnosis, and 268 had nonspecific inflammatory or non-malignant diagnoses. Twenty-three cases were non-diagnostic. Among the patients with specific diagnoses, 691 were malignant, 5 were hamartomas, 30 were fungal infections, 6 were acid-fast-positive organisms, and 40 were unspecified atypical cells. Of the patients that had a malignant diagnosis, 317 were adenocarcinoma, 197 were squamous cell carcinoma, 26 were a neuroendocrine tumor, 45 were non-small cell carcinoma (undifferentiated), 17 were small cell carcinoma, and 89 were other metastatic malignancies to the lung. Various common complications, including pneumothorax (337), hemorrhage (128), and hemoptysis (17), were observed, and 42 of the cases required chest tube intervention; others were treated with observation. Other rare complications observed included hemothorax (4) and oxygen desaturation (2), and there was no death in this series. (4) **Conclusions**: CT-guided needle biopsy is a reliable diagnostic modality for patients with lung parenchymal and pleural nodules, and it can effectively distinguish between benign and cancerous lesions before invasive procedures such as video-assisted thoracoscopy (VATs) or thoracotomy are planned. Our study showed a higher rate of pneumothorax and pulmonary hemorrhage compared to the rates established in guidelines, attributable to the varying experience level in a busy training academic center.

## 1. Introduction

Computed Tomography (CT)-guided lung biopsy was first introduced in 1965 to diagnose retroperitoneal mass [1,2] and has since been pivotal in averting unnecessary invasive lung procedures, such as thoracotomies and wedge resections [3]. Globally, lung cancer ranks among the leading causes of cancer-related mortality in men and women [3,4]. Therefore, an accurate and timely diagnosis through needle biopsy is paramount, particularly to initiate appropriate and timely treatments.

The exponential rise in the recommendation of lung cancer screening among high-risk individuals employing low-dose CT has accentuated the demand for subsequent CT-guided lung biopsy [5]. This procedure is widely accepted for evaluating lung nodules [5,6], with minimal risk compared to surgical interventions [5]. It is worth noting that the reported complication rates for CT-guided lung biopsy show substantial variability. Some studies highlight a higher complication rate with core needle biopsy (CNB) relative to fine needle aspiration biopsy (FNAB) [7]. In contrast, others fail to establish a significant difference between the two modalities [7]. In various studies, CNB and FNAB are frequently employed in tandem [8], with FNAB typically utilized for cytology examinations and CNB for histopathological evaluation [3].

Transthoracic CT-guided lung biopsy is highly recommended for chest wall or peripheral lesions due to easy access and limited lung parenchymal penetration [3]. Other diagnostic procedures for evaluating lung nodules include transbronchial biopsy, bronchoscopy, washing and brushing cytology, mediastinoscopy, and thoracotomy. Although many of these techniques are invasive, their selection depends on the precise location of the lung lesion, making them indispensable for the histopathological diagnosis of specific lung nodules [3].

In the decision-making process for selecting a diagnostic procedure, two key factors must be weighed: complication rate and diagnostic yield. Consequently, to assess the role of CT-guided needle biopsy in evaluating lung nodules, it is imperative to ascertain its safety and diagnostic efficacy. In this study, conducted as a single-center experience, we sought to determine the diagnostic and complication rates of CT-guided core needle biopsy in diagnosing lung nodules and to compare our findings with established rates in the literature. Most published studies regarding the diagnostic and complication rate of CT-guided needle biopsy for diagnosing lung lesions have small sample sizes and were carried out outside the United States, with Europe and Asia being the dominating regions. Our research has a larger sample size and was conducted in a busy academic center in the United States.

Our study shows a remarkable diagnostic yield, above the recommended rate by the Society of Interventional Radiology (SIR) guideline. However, our complication rate was above the acceptable rate established by the SIR, with pneumothorax and pulmonary hemorrhage comprising most of our complications. Interestingly, while the complication rate in our study falls short of the acceptable rate in the guidelines, a study by Huo et al. showed a higher rate of pneumothorax complication in the North American region [9]. Hence, this merits investigating whether the complication rate, particularly pneumothorax, for diagnosing lung lesions using CT-guided needle biopsy varies across regions and among United States’ academic training hospitals. Our research is the first to pose this inquiry.

## 2. Materials and Methods

This retrospective study received approval from the Institutional Review Board (IRB), which waived the requirement for informed consent. We retrospectively reviewed CT images and biopsy records of 1063 patients (532 males [50.5%], 531 females [49.95%]) with a mean age of 64.6 years (age range: 21 to 92 years) who underwent CT-guided needle biopsy of pulmonary parenchyma and pleural lesions within our healthcare system between 2016 and 2020. Bronchoscopy was not a feasible or appropriate option for these patients.

Exclusion criteria for this study encompassed CT-guided needle biopsies involving mediastinal lesions, with our study’s primary emphasis on lung parenchymal and pleural lesions. Additionally, cases of CT-guided needle biopsy aborted at any point during the procedure were excluded from our analysis.

**Figure 1:**
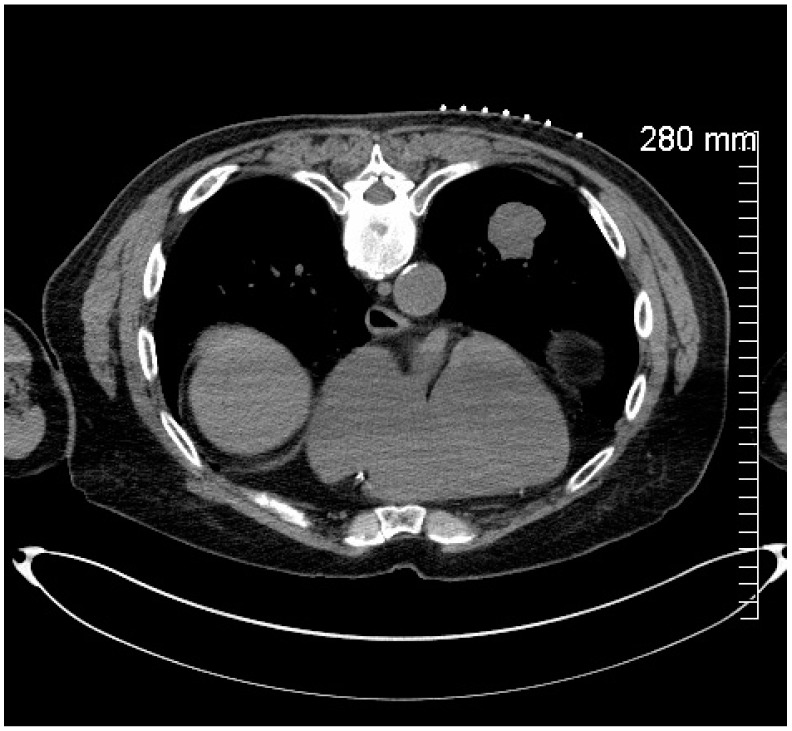
Pre-procedure CT chest without contrast demonstrating a solitary 3.2 × 3.1 cm left lower lobe lung mass in an 82-year-old male with a history of scalp melanoma pre-senting with solitary 3.2 × 3.1 cm left lower lobe lung mass.

**Figure 2:**
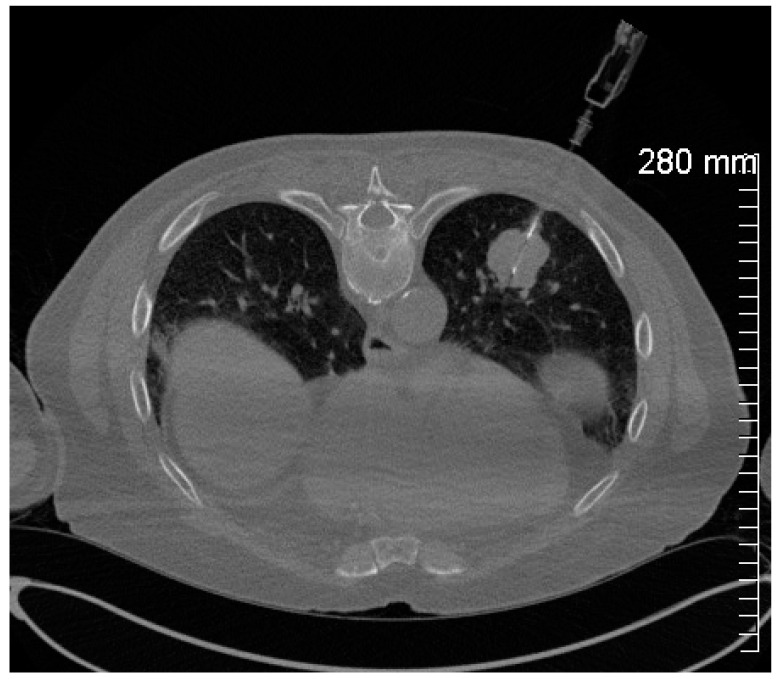
Intraprocedural CT fluoroscopic image demonstrating a 20-gauge core needle biopsy of the left lower lobe mass.

Procedure technique: The patient was positioned on the CT table, and an initial scan was conducted. Under the precise guidance of CT imaging, access to the lesion was achieved using a 17- or 19-gauge coaxial needle system. Biopsy samples were obtained through the coaxial needle, employing an 18- or 20-gauge biopsy needle, as shown in Figure 1 and Figure 2 below. Once sampling was deemed sufficient, the needle was carefully withdrawn, and an occlusive dressing was applied.

In cases where Fine Needle Aspiration (FNA) was performed, a 21- or 22-gauge needle was employed; at least one to two FNA needle passes were conducted in conjunction with core needle biopsy (CNB), and numerous needle passes were made, in rare cases, when FNAB was performed independently. In most cases, cytopathology was available during the procedure.

Afterward, post-procedural CT images were obtained to assess potential complications. Patients were then transferred to the recovery room. Routinely, chest radiographs are obtained in an hour or two hours post-biopsy to monitor for developing pneumothorax.

Histological Characterization: In our study, specimens acquired through Core Needle Biopsy (CNB) were dedicated to histological evaluation, whereas samples obtained via Fine Needle Aspiration (FNA) were sent for cytological assessment. The diagnostic outcomes of the biopsies were classified into three categories: malignant (including primary or non-primary lung carcinomas), benign (specific or nonspecific), or non-diagnostic. A result was categorized as non-diagnostic if the sample was deemed insufficient for a conclusive diagnosis by the pathologist.

## 3. Results

Table 1 presents a comprehensive overview of the patient’s demographics, lesion characteristics, and biopsy details. Our retrospective analysis included the medical records of 1063 patients who underwent CT-guided needle biopsies for parenchymal or pleural lung nodules. Among these patients, 246 were current smokers, 229 were past smokers, 147 were non-smokers, and 441 had unspecified smoking history. The study cohort consisted of 532 males and 531 females, with a mean age of 64.6 years and an age range from 21 to 92 years.

Tumor size was available in 385 patients, while 678 patients lacked a documented tumor size. Among the 385 patients with recorded tumor size, the mean lesion diameter was 1.89, with a median of 1.6 cm and a range extending from 0.26 cm to 9.2 cm. The number of needle passes varied from 1 to 14, with a mean of 4 needle passes and a mode of 4 needle passes. Needle passes exceeding nine were noted in only 15 patients, and the number of needle passes remained unrecorded for 25 patients. Core Needle Biopsy (CNB) was the predominant biopsy procedure performed in this study, which was conducted in 934 patients. Fine Needle Aspiration (FNA) was performed independently in 12 patients, and a combination of CNB and FNA was undertaken in 116 patients.

Additionally, 15 patients met the study criteria but were excluded due to the abortion of their CT-guided needle biopsy procedures at some point during the procedure. Aborted procedures were mainly due to the inability of the patient to maintain still body motion under local anesthesia.

The diagnostic yield was calculated by adding the number of cases with a definitive diagnosis and dividing the obtained value by the total number of cases in the sample. Definitive diagnosis through adequate sampling was successfully obtained in 1040 cases, constituting an impressive 97.84% of the study cohort, resulting in histological diagnosis. Among the 1040 patients with diagnosis, 772 cases had specific diagnoses, which included 602 malignant cases, 40 unspecified atypical cells, 89 lung metastasis, 30 fungal infections, six mycobacteria (acid-fast positive organisms), and 5 hamartomas. The remaining 268 diagnosed cases received inflammatory/non-malignant diagnoses from the pathologist. Nevertheless, in 23 cases, the obtained samples were deemed non-diagnostic due to their inadequacy, as determined by the pathologist. The results of malignant and benign lesions are detailed in Table 2 below.

The predominant diagnoses from successful biopsies were cancerous, accounting for 602 (65.0%) cases. Notably, adenocarcinoma of the lung emerged as the most prevalent cancer subtype, representing 29.82% of the diagnoses. Squamous cell carcinoma (SCC) followed closely at 18.53%, while non-small cell carcinoma was identified in 4.23% of cases. On the other end of the spectrum, primary lung tumor diagnoses of neuroendocrine tumors and small cell carcinomas were less frequent, constituting 2.45% and 1.6% of cases, respectively.

Among benign specific lung lesions, fungal organism infections were the most diagnosed, occurring in 2.82% of cases. Other benign-specific lesions included mycobacterium and hamartomas, with diagnostic rates of 0.56% and 0.47%, respectively.

A subset of benign lesions, classified as benign nonspecific, were characterized by pathologists as atypical cells without a specific diagnosis. This category comprised 3.76% (40 patients) of the study.

The overall complication rate was found by summing together the subgroups of complications and dividing the obtained value by the total number of cases in the sample. In our study, complications were observed in 488 patients, resulting in an overall incidence rate of 45.91%. Pneumothorax (PTX) was the most prevalent complication, affecting 337 (31.7%) patients. Other complications, including hemorrhage, hemoptysis, and hemothorax (HTX), exhibited incidence rates of 12.04% (128 patients), 1.6% (17 patients), and 0.38% (4 patients), respectively. Table 3 below outlines the details of complication rates.

Among the 488 patients who experienced complications, 60 necessitated further interventions. Specifically, 42 patients (3.95%) required chest tube placement, 13 patients (1.22%) underwent needle aspiration, and 5 patients (0.47%) required hospitalization in the Intensive Care Unit (ICU). No deaths were recorded among any of the patients.

## 4. Discussion

Lung cancer is the most prevalent cancer among men and ranks second in frequency among women, marked by its high mortality rate [3]. As a result, the prompt evaluation of lung lesions becomes imperative. The advent of high-resolution CT scans, with enhanced sub-millimeter capabilities, has substantially increased the detection rates of small pulmonary nodules, including early-stage peripheral lung cancers [5]. Consequently, percutaneous lung needle biopsy has emerged as a simple, safe, and highly accurate clinical procedure [2,5]. Notably, needle biopsy for diagnosing lung nodules is less invasive, typically obviating the need for general anesthesia [5]. This technique is an alternative to video-assisted thoracoscopic surgery (VATS) for diagnosing pulmonary nodules [5]. Another alternative is the Endobronchial Ultrasound-Guided Transbronchial Needle Aspiration (EBUS-TBNA); however, it often exhibits a lower sensitivity rate than CT-guided needle biopsy [5].

Across the existing body of literature, CT-guided needle biopsy’s diagnostic efficacy and precision for pulmonary lesions have been documented to range from 65% to 96% [10]. In their 2020 quality improvement guideline, the Society of Interventional Radiology (SIR) noted diagnostic success spanning from 92% to 96.4%, with a recommended Quality Improvement (QI) threshold falling between 85% and 90% [11]. This significant shift in the minimum acceptable diagnostic rate in the 2020 guideline, as compared to the 2010 guideline, where the diagnostic yield was set at 77% [12], can largely be attributed to the advent of precision medicine [11]. The 2010 American College of Radiology (ACR) and SIR guidelines stipulate a diagnostic yield threshold of at least 75% for thoracic and pulmonary lesions, typically achievable with 1 to 3 needle passes [10,12]. In contrast, the 2020 SIR guideline reported a diagnostic success rate of 93.1% ± 5.4% for all percutaneous needle biopsies (PNB), with a minimum diagnostic threshold of 82% [11].

Our study examined the diagnostic and complication rates in a cohort of 1063 patients who underwent CT-guided needle lung biopsies. Our study primarily used CNB, with limited utilization of FNA, while few incorporated both CNB and FNA. Remarkably, we achieved an outstanding diagnostic rate of 97.84%. A non-diagnostic result, indicated by the pathologist when the sample was deemed insufficient for a conclusive diagnosis, was observed in only 2.16% of cases. This outcome closely aligns with the diagnostic and non-diagnostic rates reported in the existing literature. For instance, Huang et al. reported an overall diagnostic yield of 93.9% in 198 patients, categorizing them into two groups based on lesion size [13]. Notably, their study showed a diagnostic yield of 83.7% for patients with lesions measuring less than 15 mm and an impressive yield of 96.8% for those with lesions exceeding 15 mm [13].

Our study encompassed a spectrum of tumor sizes, ranging from 0.26 cm to 9.2 cm, with a mean size of 1.89 cm and a median of 1.6 cm among the 385 out of 1063 patients for whom lesion sizes were documented. The diagnostic yield in our study most closely resembles the diagnostic rate observed in the group with lesions greater than 15 mm, as reported by Huang et al. [13] Consequently, the slightly higher diagnostic rate observed in our study, compared to others, could be partly attributed to the larger tumor sizes present in our sample, given our minimum size threshold of 0.26 cm.

In a single-center study in Istanbul, Turkey, CT-guided core needle biopsy had 90–91% diagnostic accuracy rates and a non-diagnostic yield of 9–10% [14]. A study by Takeshita et al. reported a diagnostic yield of 98.6% and an accuracy rate of 92.9%, confirmed by surgical histopathology, revealing a non-diagnostic rate of only 1.3% [15]. It is important to note that while our study did not ensure histopathological diagnostic yield through surgery, Takeshita et al. did identify a false-negative rate of 6.8% within the diagnostic failure group and an overall diagnostic failure rate of 8.3%. Therefore, among the 31.14% of patients classified as benign-nonspecific or non-diagnostic in our study, there exists an approximately 6.8% probability of a false-negative outcome and a 0.13% likelihood of a false-positive result, as revealed by Takeshita et al. [15].

Our investigation also delved into the complication rates associated with CT-guided lung biopsy. The Society of Interventional Radiology (SIR) Guidelines categorize complications into minor and major forms. Minor complications include pneumothorax that does not require intervention, pulmonary hemorrhage around the target site, and hemoptysis with spontaneous hemostasis [11,12,13]. Significant complications include pneumothorax requiring intervention, evidence of hemothorax, needle tract seeding, air embolism, and death [13].

The overall complication rate in our study was 45.9%. Takeshita et al. reported an overall complication rate of 20%, ref. [14] significantly lower than the rate we found in our research. Our higher complication rate may be attributed to carefully considering all potential complications, including minor and insignificant pneumothorax or hemorrhage, which could have inflated the overall complication rate, suggesting that our study accounted for a higher incidence of minor complications.

Pneumothorax and pulmonary hemorrhage are the most frequently reported complications in the literature, with rates varying from 4.3–52.4% [9], 9–54% [10], 15–62% [13], and 8–69% [15] for pneumothorax and 30–65.6% [15] for pulmonary hemorrhage. Our study also found pneumothorax and pulmonary hemorrhage as the most frequent complications, consistent with the findings in the existing literature [7,14]. The rate of pneumothorax in our study was 31.70%, and the rate of pulmonary hemorrhage was 12.04%. SIR reported an acceptable rate of pneumothorax as 15.3% to 22% and pulmonary hemorrhage of 6.1% [11]; other studies reported 17% pneumothorax and 3% pulmonary hemorrhage complications [14], while 38.4% in pneumothorax and 62.1% in pulmonary hemorrhage was reported by Huang et al. [13]. Takeshita et al. reported a pneumothorax rate of 36.8% [15]. Our rate of pneumothorax and pulmonary hemorrhage complication is higher than the rate reported in the SIR guidelines and by Sahin et al. [14], but lower than the rate reported in the other studies [13,15]. A systematic review of CT-guided lung biopsies by Huo et al. reported an overall pneumothorax incidence of 25.9% and a pneumothorax rate of 30.4% in the North American region [9]. The incidence of pneumothorax in North America, as reported by Huo et al., is close to the rate we found in our study.

Other complications recorded in our study include hemoptysis, hemothorax, and severe dyspnea, with complication rates of 1.60%, 0.38%, and 0.19%, respectively. No deaths were reported. Our rate of hemoptysis complication is within the acceptable complication rate for hemoptysis (1.7–4.1%) established in the 2020 SIR guideline [11]. Among those who experienced difficulties, 5.64% required intervention in the form of needle aspiration (1.22%), chest tube placement (3.95%), or hospitalization (0.47%).

Our overall rates of complication align with some previous individualistic studies [10,13,15], but deviate from a meta-analysis by Heerink et al. that reported a complication rate of 38.8% for CNB and a rate of 24% for FNA [7]. Heerink et al. reported a pneumothorax rate of 25.3%, with 5.6% requiring intervention, pulmonary hemorrhage of 18%, and hemoptysis of 4.1% in the CNB group, with a lower rate of complications in the FNA group [7]. The overall complication rate in our study exceeded the rate reported by Henrik et al. and the 2020 SIR guideline. Also, the rate of pneumothorax in our study (31.70%) was higher than the rate reported in the systematic review (25.9%) [9] and the meta-analysis study (25.3%) [7]. However, our rates of pulmonary hemorrhage and hemoptysis were lower. The increased number of needle passes, an average of 4, mode of 4, and 117 patients with greater than six passes found in our study are possible explanations for the higher-than-expected pneumothorax rate. Our slightly higher-than-expected pneumothorax rate could also result from using a large gauge needle during the procedure and targeting lesions ≤ 1 cm in size at our center. According to Table 4 below, 3.86% of pneumothorax complications arise from lesions ≤ 1.0 cm. We consider this 3.86% significant, primarily because most of the lesion size (16.84%) with known pneumothorax complications could not be obtained. As a large referral academic center, our interventional radiologists frequently attempt the biopsy of lung lesions in difficult locations or adjacent to critical structures in a cohort of patients who may not otherwise be considered candidates for percutaneous biopsy; this can also explain our higher complication rate.

In the literature, the intervention rate for pneumothorax ranges from 0–15% [9], 4.3–7.3% [7], and 7–15% [15]. Our overall intervention rate for this study is 5.64%, with chest tube placement (3.95%) and needle aspiration (1.22%) comprising the majority of the intervention modality, while hospitalization (0.47%) was the minor intervention modality. Hence, irrespective of our higher pneumothorax rate, most patients with complications in our study did not require intervention.

Finally, we identified adenocarcinomas and squamous cell carcinomas as the most diagnosed primary lung cancers, consistent with most literature sources [3,6,16,17]. In contrast to our research and prevailing publications, Gorgulu et al. reported non-small cell carcinoma and malignant epithelial lesions as the most prevalent lung cancer diagnoses in their study [3]; adenocarcinoma ranked as the second most frequent diagnosis with an incidence of 16.9%, while squamous cell carcinoma was the third most frequently diagnosed, with an incidence rate of 12.3% [3]. Across various studies, and congruent with our findings, small cell carcinoma consistently emerged as the least frequently diagnosed primary lung cancer [3,6,16,17], with incidence rates of 1.60% in our study, 0.7% according to Zhao et al., 2.96% as reported by Y. Li et al., and 6.2% by Gorgulu et al. The incidence of lung metastasis displayed variability in the literature, ranging from 2.9% [16] to 19.3% [17] among the studies we reviewed. In our study, the rate of metastasis to the lung was 8.37%, falling within the reported range in other studies. Additional diagnostic findings from our research and their respective rates are detailed in Table 2.

Our study possesses several notable strengths. It is one of the first extensive investigations into the diagnostic efficacy of CT-guided needle biopsy for pulmonary nodules conducted on a large scale. However, certain limitations warrant consideration. Firstly, our study needs comprehensive statistical analysis, which could offer more robust insights into the observed outcomes. Additionally, the study was conducted within a large academic institution with a diverse team of interventional radiologists and trainees with varying experience levels. This diversity in expertise may have influenced both the diagnostic and complication rates observed in our study. In future research, a comparative study from other academic institutions could help shed light on pneumothorax complications. Based on the findings, this could determine if the literature and guidelines underestimated the pneumothorax rate for a CT-guided biopsy of lung lesions. Ultimately, this would lead to universal quality improvement across institutions to ensure patient safety.

Also, our study did not explore the underlying factors contributing to the complications, which is particularly noteworthy due to our institution’s slightly elevated overall complication rate. Huo et al., in their systematic analysis review, noted multiple pleural punctures, large needle gauge, a medical history of emphysema, and smaller and deeper lesions as possible causes of pneumothorax during CT-guided lung biopsy that are worthy of exploring in future research [9].

## 5. Conclusions

Our study is the first to utilize a large sample size to evaluate the diagnostic yield and rate of complication in CT-guided needle biopsy. Overall, the diagnostic yield in our study was slightly above the maximum diagnostic rate reported in the literature. Our complication rates were mostly consistent with the results reported in the literature, except for our pneumothorax rate, and the rate of chest tube placement remained low. CT-guided needle biopsy is deemed a reliable modality for diagnosing lung cancer, with an acceptable complication rate. However, given the increased pneumothorax rate noted in our study, comparing our complication rates with those of other academic centers would be beneficial. This comparison would help us accurately assess our complications relative to other institutions and determine whether the complication rate for CT-guided needle biopsies for lung lesions is underestimated in academic training centers. A CT-guided needle biopsy can indeed lower the need for invasive procedures by differentiating between malignant and benign lesions. As the advancement in CT imaging continues to improve, along with the experience of radiologists and cytopathologists, we anticipate a further increase in CT-guided needle biopsy diagnostic rate and a reduction in complication rate.

## Figures and Tables

**Table 1:** Patient demographic, lesion characteristics, number of needle passes.

	N (%)
Patients (All)	
Men	532 (50.5)
Women	531 (49.95)
Mean age (years)	64.6
Median age (years)	65
Range (years)	21–92
Aborted Procedure	15 (1.4)
Tumor Size	
Mean	1.89 cm
Median	1.6 cm
Range	0.26–9.2 cm
≤2.0 cm	250 (23.52)
≥2.0 cm	135 (12.70)
Size not Indicated	678 (63.78)
Lesion Side	
Right	571 (53.72)
Left	490 (46.09)
Not specified	2 (0.19)
Location of Lesion	
Upper	486 (45.72)
Middle	50 (4.70)
Lower	357 (33.58)
Unspecified	170 (16.0)
Biopsy Performed	
CNB	934 (87.86)
FNA	12 (1.13)
BOTH	116 (10.91)
UNCLEAR	1 (0.1)
Number of needle passes	
Mean	4
Mode	4
1–3 passes	315 (29.63)
4–6 passes	606 (57.01)
7–9 passes	102 (9.60)
>9 passes	15 (1.41)
Not Indicated	25 (2.35)
Smoking Status	
Smoker	246 (23.14)
Past smoker	229 (21.54)
Non-smoker	147 (13.83)
Not Indicated	441 (41.49)
Type of lesion	
Malignant	691 (65.00)
Benign	349 (32.83)
Non-diagnostic	23 (2.16)

N = Number.

**Table 2:** Histological Diagnosis.

		N (%)
Malignant	Primary Lung Carcinoma	
	Adenocarcinoma	317 (29.82)
	Squamous Cell Carcinoma	197 (18.53)
	Non-small cell carcinoma	45 (4.23)
	Small Cell carcinoma	17 (1.60)
	Neuroendocrine tumor	26 (2.45)
	Subtotal	602 (56.63)
	Other	
	Lung Metastasis	89 (8.37)
	Unspecified atypical Cells	40 (3.76)
	Subtotal	129 (12.13)
Benign	Benign-Specific	
	Fungal organisms	30 (2.82)
	Mycobacterium (Acid Fast positive)	6 (0.56)
	Hamartomas	5 (0.47)
	Subtotal	41 (3.86)
	Benign Nonspecific	
	Inflammation/non-malignant	268 (25.22)
Non-Diagnostic	Insufficient sample	23 (2.16)
Total		1063

**Table 3:** Complications.

		N (%)
Procedure Complications	Pneumothorax	337/1063 (31.70)
	Hemothorax	4/1063 (0.38)
	Hemoptysis	17/1063 (1.60)
	Hemorrhage	128/1063 (12.04)
	SOA/Desaturation	2/1063 (0.19)
	Death	0
	Total	488/1063 (45.91)
Interventions	Chest tube	42/1063 (3.95)
	Needle Aspiration	13/1063 (1.22)
	Hospital Admission/ICU	5/1063 (0.47)
	Total	60/1063 (5.64)

ICU = Intensive Care Unit.

**Table 4:** Pneumothorax Complication rate by lesion size.

Lesion Size	Rate (%)
≤1.0 cm	41/1063 (3.86)
>1.0 cm	117/1063 (11.01)
UTO	179/1063 (16.84)
Total	337/1061 (31.70)

Unable to Obtain (UTO).

## Data Availability

The data presented in this study are available on request from the corresponding author due to the patient’s privacy rule and the Health Insurance Portability and Accountability Act (HIPAA).
